# Supplementary material for: Screening for Hepatitis C Virus Reinfection Using a Behaviour-Based Risk Score among Men Who Have Sex with Men with HIV: Results from a Case–Control Diagnostic Validation Study
Source: Pathogens. 2023 Oct 16;12(10):1248. doi: 10.3390/pathogens12101248 (PMC10610046; doi:10.3390/pathogens12101248)
Supplement: Supplementary file 1 [file pathogens-12-01248-s001.zip › pathogens-2580488-supplementary.pdf]

Supplementary Materials

Supplementary Materials to:

Screening for Hepatitis C Virus Reinfection Using a Behaviour-Based Risk Score among Men Who Have Sex with Men with HIV: Results from a Case–Control Diagnostic Validation Study

Kris Hage, Marita van de Kerkhof, Anders Boyd, Joanne M. Carson, Astrid M. Newsum, Amy Matser, Marc van der Valk, Kees Brinkman, Joop E. Arends, Fanny N. Lauw, Bart J. A. Rijnders, Arne van Eeden, Marianne Martinello, Gail V. Matthews, Janke Schinkel and Maria Prins

Figure S1. Histogram of MOSAIC risk score among cases (left) and controls (right) included in the analysis from the MOSAIC study

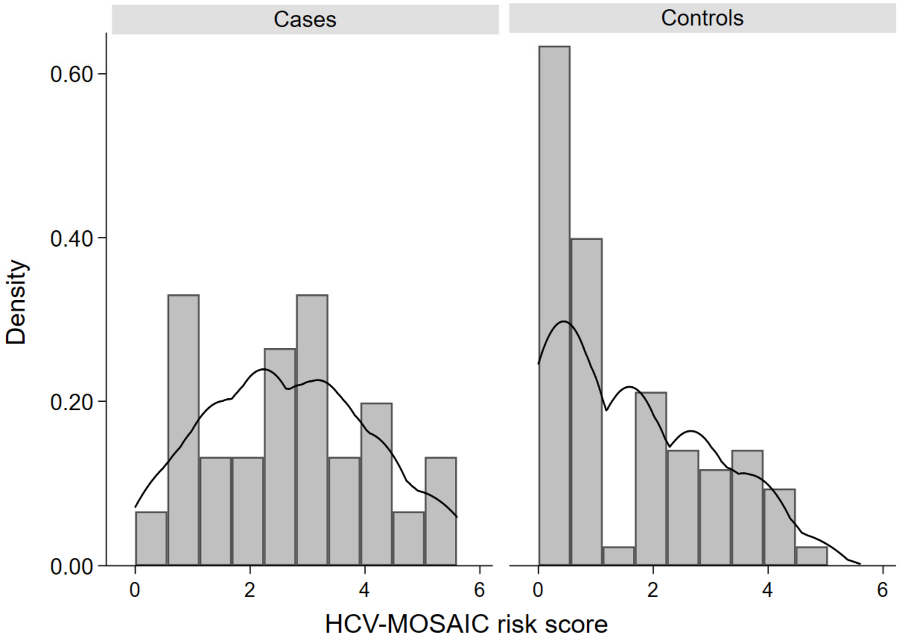

Density represents the proportion of individuals within evenly-distributed bands of the HCV-MOSAIC risk score.

Abbreviations: HCV, hepatitis C virus; MOSAIC, MSM (men who have sex with men) Observational Study of Acute Infection with hepatitis C.

Figure S2. Non-parametric receiver operating characteristic (ROC) curve of the HCV-MOSAIC risk score for reinfection in sensitivity analyses where we restricted the HCV-MOSAIC risk score in the training dataset to the same risk factors measured in the validation dataset.

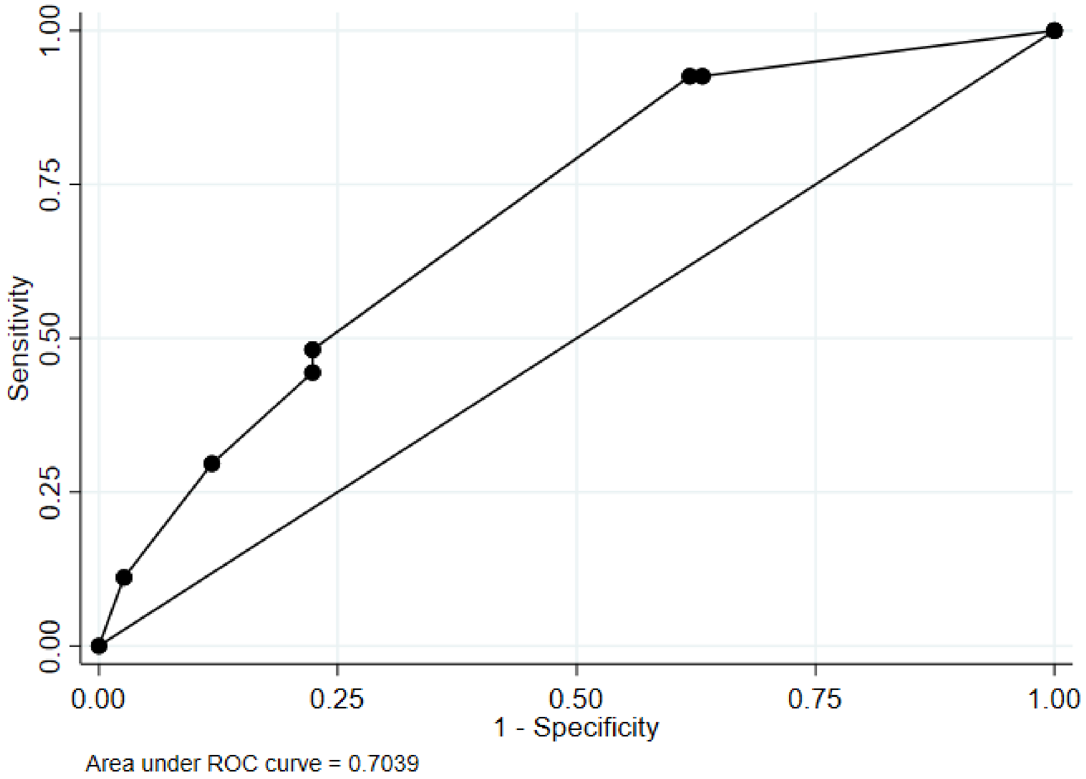

Abbreviations: HCV, hepatitis C virus; MOSAIC, MSM (men who have sex with men) Observational Study of Acute Infection with hepatitis C.
